# Stress Concentration and Damage Factor Due to Central Elliptical Hole in Functionally Graded Panels Subjected to Uniform Tensile Traction

**DOI:** 10.3390/ma12030422

**Published:** 2019-01-30

**Authors:** Wenshuai Wang, Hongting Yuan, Xing Li, Pengpeng Shi

**Affiliations:** 1 School of Mathematics and Statistics, Ningxia University, Yinchuan 750021, China; wws@nxu.edu.cn (W.W.); yhtgfky@163.com (H.Y.); 2 State Key Laboratory for Strength and Vibration of Mechanical Structures, Shaanxi Engineering Research Center of NDT and Structural Integrity Evaluation, School of Aerospace, Xi’an Jiaotong University, Xi’an 710049, China

**Keywords:** functionally graded materials, inhomogeneous composite materials, material design, stress concentration factor, failure and damage, elliptical hole, finite element method

## Abstract

Functionally graded material (FGM) can optimize the mechanical properties of composites by designing the spatial variation of material properties. In this paper, the stress distribution of functionally graded panel with a central elliptical hole under uniaxial tensile load is analyzed. Based on the inhomogeneity variation and three different gradient directions, the effects of the inhomogeneity on the stress concentration factor and damage factor are discussed. The study results show that when Young’s modulus increases with the distance from the hole, the stress concentration factor decreases compared with that of homogeneous material, and the optimal design of r-FGM is better than that of x-FGM and y-FGM when the tensile load. In addition, when the associated variation of ultimate stress is considered, the choice of scheme to reduce the failure index is related to the strength-modulus exponent ratio. When the strength-modulus exponent ratio is small, the failure index changes with the index of power-law, which means there is an optimal FGM design. But when the strength-modulus exponent ratio is large, the optimal design modulus design is to select a uniform material that maximizes the modulus at each point. These research results have a certain reference value for further in-depth understanding of the inhomogeneous design for FGM.

## 1. Introduction

Functionally graded materials (FGM) are a class of composite materials that have smooth and continuous changes in material properties, thus reducing the stress concentrations in the conventional composite materials [1]. Since the concept of FGM was proposed by researchers in the late 1980s [2], extensive research works have been carried out on it. Many review papers have systematically introduced and forecasted the different progress of FGM researches. Recently, Zhang et al. [3] introduced the development of the emerging additive manufacturing research on FGM. Xu et al. [4] reviewed the state of the art of energy absorption of FGM, and discussed the effects of the graded properties on the crashworthiness. Cramer et al. [5] proposed a review of functionally graded thermoelectric generators, which is considered to be an effective solution for the temperature bandwidth, current output range, and lifetime. Petit et al. [6] introduced the rationale for using FGM in the biomedical field, and reviewed the three main types of graded materials (eg., composition, porosity and microstructural graded ceramics). The mechanical problems of the FGM have also drawn much attention. The progress of the resistance of FGM to contact deformation and damage is reviewed by Suresh [7]. Birman [8] outlined the steps of thermoelastic analysis of FGM, from their micromechanical characterization to the structural response. The fracture studies for FGM continuum can be found in the survey by Shanmugavel et al. [9]. In addition, Jha et al. [10] published another detailed overview focused on the thermoelastic statics, vibration and stability analysis of FGM plates. Some comprehensive reviews of the developments, applications, various mathematical idealizations of materials, temperature profiles, modeling techniques and solutions methods for the thermal analysis of FGM plates are presented by Swaminathan et al [11,12]. Some scholars applied the FGM concept to the elastostatic problems and obtained some exact solutions of orthotropic inhomogeneous Saint-Venant beams and isotropic Kirchhoff plates [13,14]. In addition, some scholars applied the concept of FGM to the study of mechanical behavior of nanomaterials by using nonlocal model or gradient elasticity model [15,16].

Due to the specific functional requirements, the influences of inhomogeneous variation on the FGM properties are studied, which has an important reference value for the preparation, performance and use of FGM. Since holes or inclusions are common defects in materials, the stress concentration of FGM has been extensively studied by finite element method and analytical method. Mohammadi et al. [17] used the Frobenius series solution to analyze the effect of inhomogeneous stiffness and Poisson’s ratio on the stress concentration factor around the circular holes of infinite plates. Based on the complex function method and the conformal mapping technique, Yang and Gao [18] solved the stress concentration problem of FGM infinite plates with elliptic holes. Dave and Sharma [19] also used the complex variable function method to solve the problem of the FGM plate with rectangular holes. Based on the variable separation method, the analytical solutions of stress and strain distribution around the circular elastic inclusion and elliptical nano-fiber inclusion are obtained by Shi [20,21]. Goyat, et al [22] used the extended finite element method to analyze the stress concentration of the FGM layer in an infinite plate with a pair of circular holes under different loads. Based on the first-order shear deformation theory and Von-Karman hypothesis, Mehrparvar and Ghannadpour [23] analyzed the non-linear behavior of FGM plates with square and rectangular notches. In addition, Shi et al. [24,25,26,27] used the integral equation method to study the influence of the existence of the central circular hole on the interface fracture behavior of the FGM composite cylindrical structure. 

To reduce the stress concentration factor, Sburlati [28] studied the effect of an inhomogeneous annular made of FGM on the stress distribution around a hole in a homogeneous plate. Aiming to reduce the stress concentration factor around the notch, Gouasmi, et al. [29] used the finite element method to study the performance of the FGM layer near the notch of the ceramic plate. Sburlati, et al. [30] analyzed the effect of FGM layers on the stress concentration factor in a homogeneous plate with holes based on the finite element method. Hsu and Chien [31] combined the finite element method and image processing technology to evaluate the influence of electronic discharge machining parameters on the surface quality of the plate with holes, which can quickly evaluate the stress concentration factor. Based on the finite element method and U-transform method, Yang, et al. [32] analyzed and studied the three-dimensional stress concentration of rectangular holes. Kubair and Bhanu-Chandar [33] investigated the FGM with the elastic modulus of power law and exponential variation, and simulated the FGM plates with circular holes under uniaxial tension by the multi-parameter finite element method. Nie, at al. [34] analyzed the stress concentration of FGM plates with Young’s modulus of radial variation and Poisson’s ratio under uniaxial tension. Kim and Paulino [35] analyzed the effects of elastic modulus and Poisson’s ratio on the properties of isotropic and orthotropic FGM plates by isoparametric gradient finite element method. In addition, with consideration of the associated variation of ultimate stress, the combined optimization using both moduli and ultimate stress is studied by Huang et al. [36], and the optimization for the full spatial variation is completed by Chen et al. [37].

In this paper, the stress distribution of FGM panels with a central elliptical hole under uniaxial tension load is analyzed, and the effects of the inhomogeneous properties on the stress concentration factor (SCF), failure index and damage factor are discussed. In Section 2, the problem description is given. Two inhomogeneous variations and three different gradient directions are proposed here. In Section 3, the stress problems due to a central elliptical hole for FGM with different forms of elastic modulus and different gradient direction are calculated, and the influences of the inhomogeneous characteristics of FGM on stress concentration, failure index and damage factor are analyzed in detail. The conclusion for this paper is given in Section 4.

## 2. Problem Description

### 2.1. Problem Description

**Figure 1:**
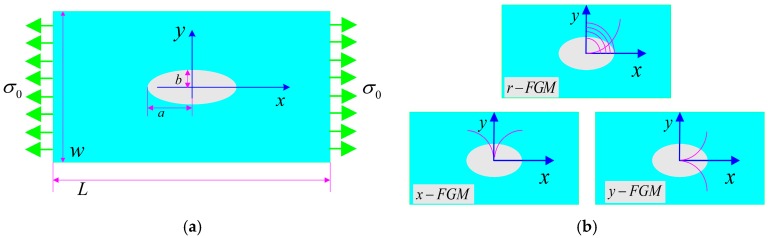
Schematic sketch of boundary value problem (**a**) a rectangle panel with an elliptical hole subjected to a uniform tensile traction; (**b**) three different gradient directions. The origin of the Cartesian and polar coordinates coincides with the center of the elliptical hole. In the case of the r-, x- and y-FGM symmetric property variations are shown.

We consider an isotropic and linearly elastic FGM panel with a central elliptical hole subjected to a uniform tensile traction, as shown in Figure 1a. The length and width of the rectangular panel are *L* and *W*, respectively, and the semi-major/semi-minor axes of the ellipse hole are *a* and *b*, respectively. In this paper, a finite-size rectangular panel is selected, and the left and right end are subjected to a tensile load $\sigma_{0}=P/\left(tW\right)$, where *P* is the value of the force, *t* is the thickness of the panel. Here, we use cylindrical coordinates (*r*, *θ*) and Cartesian coordinates (*x, y*) with origin at the hole center to describe this problem. 

### 2.2. Inhomogeneity Variation

Previous studies show that the effect of varying Poisson ratio on the stress distribution is negligible [34]. In this paper, we assume Poisson’s ratio to be constant and set to be v=0.25. 

In the study of mechanical problems of FGM, the problem is often analyzed by assuming that the material parameters satisfy a certain function form, which can simplify the complexity of the problem. In order to discuss the effect of the inhomogeneity of the FGM, we assume that the nodal values of Young’s modulus satisfies the power-law inhomogeneous variation:

(1) $$E\left(\phi \right)=E_{ref}\left[1+\gamma {\left(\phi /Lg\right)}^{c}\right],$$

where $E_{ref}$ is the reference value of Young’s modulus, γ is the modulus ratio, *c* is the index of power-law variation, Lg is the inhomogeneity length scale, ϕ is a simple function of (*x, y*).

In order to study the effect of different gradient directions, we assume the following forms for the function ϕ.

(2) $$\phi =\left\{ \begin{array}{l} \sqrt{x^{2}+y^{2}}\\ x\;\mathrm{or}\;\left|x\right|\\ y\;\mathrm{or}\;\left|y\right| \end{array}\right.,$$

and three different gradient directions of FGM are shown in Figure 1b.

For the power-law inhomogeneous variation, the gradient variation reflected by Equation (1) can be divided into the following two cases according to the different values of parameter c. 

Case 1: When c>0, for a finite panel problem, *Lg* can be set to half length of the rectangular panel. The material parameters at the center of the circle are satisfied:

(3) $$E_{0}=E\left(0\right)=E_{ref},$$

and:

(4) $$E\left(\phi \right)=E_{0}\left[1+\gamma {\left(\phi /Lg\right)}^{c}\right]$$

This gradient variation Equation (4) is consistent with the power-law gradient variation given in [33].

Case 2: When c<0, *Lg* can be set to be the semi-minor axis of the elliptical hole. For an infinite panel problem, the material parameters at infinity point satisfies:

(5) $$E_{\infty }=E\left(\infty \right)=E_{ref},$$

and:

(6) $$E\left(\phi \right)=E_{\infty }\left[1+\gamma {\left(\phi /a\right)}^{c}\right],$$

when ϕ=r, this proposed gradient variation is consistent with that given in [34].

The above analysis shows that the gradient variation given in Refs. [33,34] can be unified by the gradient variant expressed by Equation (1) in this paper.

### 2.3. Stress Concentration and Damage Factor

Here, we calculate the stress concentration of FGM with a central elliptical hole under uniaxial tension with different inhomogeneous parameters. Stress concentration factor *K* is defined as $K=\sigma_{\max}/\sigma_{nom}$, where $\sigma_{\max}$ is the maximum value of stress component along the x direction in a panel, and $\sigma_{nom}=\frac{P}{t(W-2r)}=\frac{\sigma_{0}W}{t(W-2r)}$ is the reference of stress value.

For some materials, its elastic modulus and strength change with varying porosity and material density [36]. Among them, the strength and elastic modulus of the material satisfy the following relationship.

(7) $$\sigma_{allow}=C_{0}E^{\delta },$$

where factor δ is called the strength-modulus exponent ratio, and $\sigma_{allow}$ is the limit strength or maximum allowable stress at that point.

By considering the associated variation of ultimate stress, a failure index Φ that accounts for both strength and stress is used for design purposes. Referring to the results of Ref. [36], the failure index can be defined as follows:

(8) Φ=max{ψ(x,y)},(x,y)∈Ω,

where:

(9) $$\psi =\frac{\max\left(\left|\sigma_{1}\right|,\left|\sigma_{2}\right|\right)}{\sigma_{0}{\left(E/E_{ref}\right)}^{\delta }},$$

where $\sigma_{1}$ and $\sigma_{2}$ are the principal stresses at an arbitrary point in the panel, and $\sigma_{0}=P/\left(tW\right)$ is the value of the tensile stress.

## 3. Results and Discussions

In this paper, the finite element method is introduced for the mechanical analysis of FGM. In order to describe the numerical simulations clearer, the parameter values used for in the following numerical simulations are given in Table 1.

### 3.1. Verification

Here, the comparisons between the proposed results and other results obtained in the previous researches are given to verify the correctness of the calculation program used in this paper.

#### 3.1.1. Verification 1: Analysis of Homogeneous Rectangular Panel with a Circular Hole

The stress concentration factors of the homogeneous rectangular panel with a central circular hole are analyzed. The stress concentration factors of a central single circular hole in finite width and infinite length panel can be calculated for tension load by the following theoretical formula [38]:

(10) $$K=3-3.14\times 2a/W+3.667\times {\left(2a/W\right)}^{2}-1.527\times {\left(2a/W\right)}^{3}.$$

**Figure 2:**
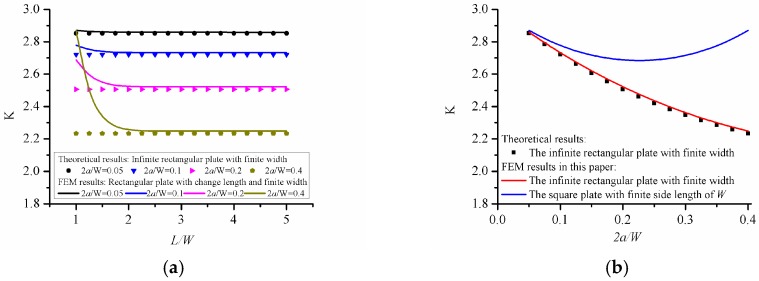
Variation of stress concentration factors for a central circular hole in a rectangular panel (**a**) rectangular panels with different lengths, (**b**) rectangular and square panels.

Figure 2a shows the comparison between the results from finite element method and the analytical results. It can be seen that as the length of the rectangular panel increases, the result of stress concentration factors gradually decrease and tend to be stable. When *L/W* exceeds 3, the results from finite element method for the finite-length rectangular panel are equal to the analytical results for the infinite length rectangular panel. In particular, the numerical solution of the rectangular panel with *L/W* = 5 and the square panel with *L/W* = 1 are given in Figure 2b. It can be seen that the numerical solution of the rectangular panel with *L/W* = 5 is consistent with the analytical result of the infinite length rectangular panel. The results of the square panel decrease first and then increase as the width increases, which has the difference between the results of the finite-size square panel and the results of the infinite rectangular panels. In general, this comparison can prove the correctness of the present calculation program.

#### 3.1.2. Stress Analysis of FGM Panel without Hole

**Figure 3:**
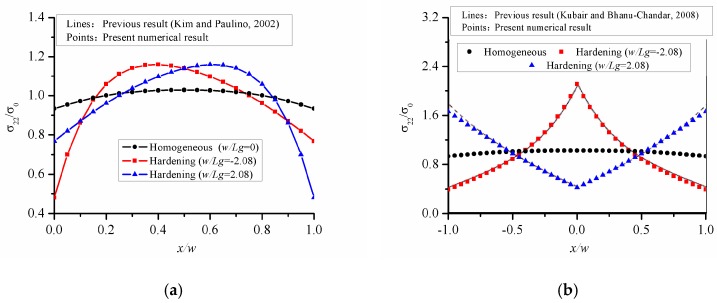
Variation of the normal stress in a uniform FGM panel without hole under tension load along the *y*-direction. (**a**) x-FGM changes monotonously along *x*-direction; (**b**) x-FGM symmetrical about x = 0.

Based on the triangular element, the stress concentration problem of an FGM square panel, without a hole, is solved under uniaxial tension along the *y*-direction, and then the normal stress distribution is calculated. As shown in Figure 3, the stress results are normalized by referring to the value of external tension load $\sigma_{0}$ along the *x*-direction. The normalized normal stress for the two configurations is shown in Figure 3. Firstly, the problems raised by Ref. [35] is recalculated and the results are shown in Figure 3a. Here, a softening material means that Young’s modulus is progressively decreasing away from the origin of the coordinates, and the hardening material means that Young’s modulus gradually increases away from the origin of the coordinates. In this problem, the origin is located at the left or right end of the panel, Young’s modulus adopts the exponentially variation and makes it varies along the x direction. The expression of Young’s modulus is $E\left(x\right)=E_{0}\exp\left(x/L_{g}\right)$ and the size of the panel satisfies w/Lg=±2.08. When the origin is located at the center of the panel, the problem proposed in Ref. [33] is resolved in Figure 3b. The expression of Young’s modulus is $E\left(x\right)=E_{0}\exp\left(\left|x\right|/Lg\right)$ and the size of the panel satisfies w/Lg=±2.08. As can be seen from Figure 3, the normal stress of the homogeneous panel without hole hardly changes with the change of the position of the x. For FGM panel, even if there is no circular hole defect, there is an inhomogeneous stress distribution in the panel. In Figure 3a, as Young’s modulus varies monotonously along x, the stress first increases and then decreases, or first decreases and then increases. Young’s modulus discussed in Figure 3b is symmetrical with respect to x = 0, so the distribution of normal stress is symmetrical along the x-axis and the maximum/minimum normal stress appears in the center of the panel. Moreover, the stress variation shown in Figure 3 varies smoothly and satisfies the global equilibrium in an integral sense as $\int \left(\sigma_{22}/w\right)dx=\sigma_{0}$. In general, the results in this paper are consistent with those in Refs [33,35], which proves the feasibility of the present calculation program.

#### 3.1.3. Stress Analysis of FGM Panel with a Circular Hole

**Figure 4:**
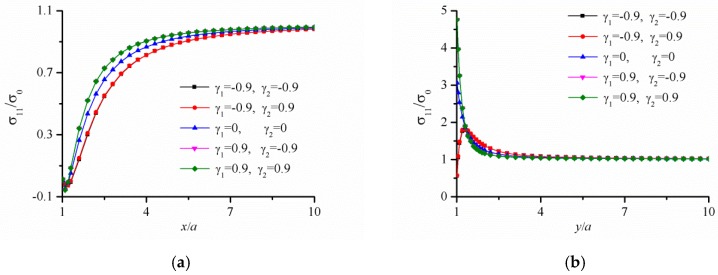
Variation of the (**a**) radial stress on the line y = 0, (**b**) the hoop stress on the line x = 0 in a uniform FGM panel with a circular hole under tension load along the *x*-direction.

The stress distribution near the hole of FGM panel with a circular hole under tension load along the *x*-direction are recalculated which is given in Ref. [34]. Young’s modulus in this analysis is $E\left(r\right)=E_{ref}\left[1+\gamma_{1}{\left(r/a\right)}^{c}\right]$, Poisson ratio is $v\left(r\right)=v_{ref}\left[1+\gamma_{2}{\left(r/a\right)}^{c}\right]$, and c=−5. Figure 4 shows that when the value of $\gamma_{1}$ is positive, the hoop stress on the hole surface reaches the maximum value at the point of *x* = *a*. Correspondingly, when the value of $\gamma_{1}$ is negative, the hoop stress on the hole surface reaches the minimum value at the point of *x* = *a*. However, the values of $\gamma_{2}$ have little influence on the stress results. In addition, the hoop stress decreases gradually as it moves away from the circular hole. When the value of y/a is close to 5, the stress reaches a stable value. As shown in Figure 4, the new calculation results in this paper are in good agreement with those given in Ref. [34], which confirms the reliability of the present calculation program.

### 3.2. Stress Concentration Factor

#### 3.2.1. The Power-Law Inhomogeneous Variation When c > 0

**Figure 5:**
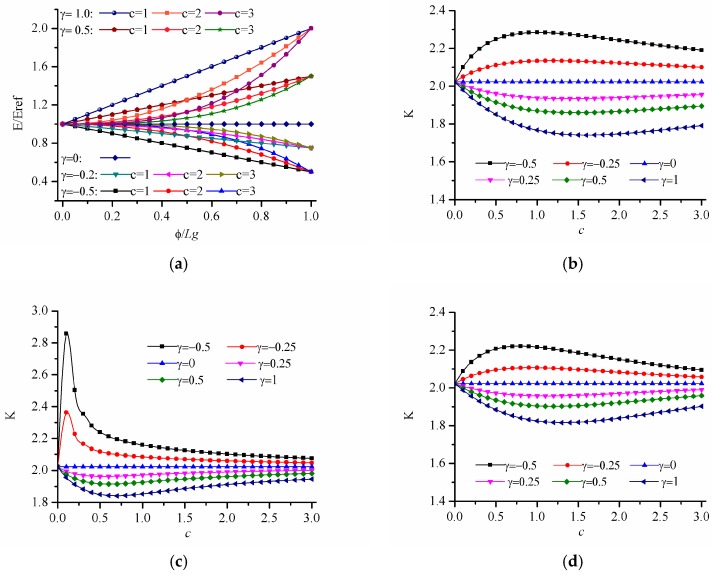
Variation of stress concentration factor K for power-law inhomogeneous variation when c > 0. (**a**) variation of Young modulus; (**b**) results for r-FGM; (**c**) results for x-FGM; (**d**) results for y-FGM.

**Figure 6:**
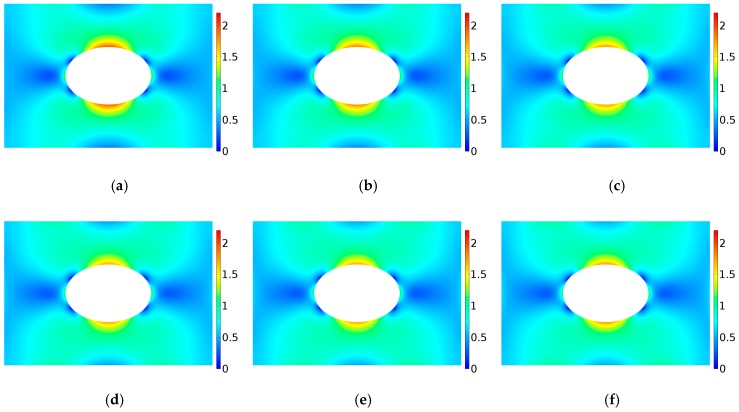
Variation of dimensionless Von Mises stress distribution for r-FGM when γ=1; The power-law inhomogeneous variation when c < 0. γ=−0.5. (**a**) γ = 1, c = 0.1; (**b**) γ = 1, c = 0.5; (**c**) γ = 1, c = 1; (**d**) γ = 1, c = 1.5; (**e**) γ = 1, c = 2.0; (**f**) γ = 1, c = 3.

Here, we calculate the stress concentration of FGM with a circular hole under uniaxial tension. Young’s modulus varies in the power-law form as $E\left(\phi \right)=E_{0}\left[1+\gamma {\left(\phi /Lg\right)}^{c}\right]$, where c>0. By changing the values of c and γ, the variation trend of stress concentration factor is obtained. Figure 5a depicts the variations of Young’s modulus $E/E_{ref}$ with ϕ/Lg under different gradient control parameters where γ=−0.5,−0.25,0,0.5,1 and c=1,2,3. When γ=0, it satisfies $E=E_{0}$, which corresponds a homogeneous panel. When γ>0, $E/E_{ref}$ gradually increases with the increase of ϕ/Lg. When γ<0, $E/E_{ref}$ gradually decreases with the increase of ϕ/Lg. Figure 5b–d gives the stress concentration factor when the elastic modulus changes along the directions of r, x and y, respectively. As shown in Figure 5b–d, when γ>0, the stress concentration factor K decreases first and then increases with the increase of c, and when γ<0, the stress concentration factor K increases first and then decreases with the increase of c. In addition, it can be seen that when γ>0, the stress concentration factor can be reduced compared with that of homogeneous materials. Since the corresponding stress concentration factor becomes minimum when γ=1, the dimensionless Von Mises stress distribution are given in Figure 6. It can be seen that the maximum value of the dimensionless Mises stress first decreases and then increases with the increase of *c*. The means there exists an optimal value of power law index because the stress distribution does not change monotonously with the increasing power law index. 

#### 3.2.2. The Power-Law Inhomogeneous Variation When c < 0

**Figure 7:**
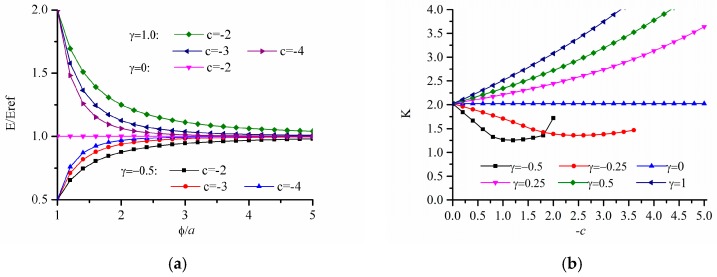
Variation of stress concentration factor K for power-law inhomogeneous variation of r-FGM when c < 0; (**a**) variation of Young’s modulus; (**b**) results for r-FGM.

**Figure 8:**
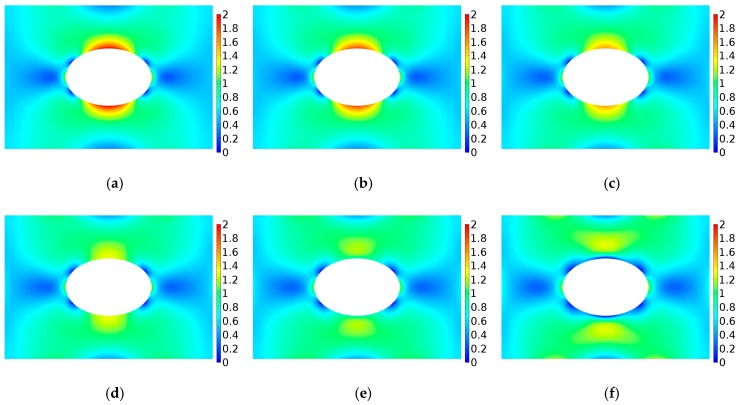
Variation of dimensionless Von Mises stress distribution for r-FGM when γ=−0.5. (**a**) γ = −0.5, c = 0.0; (**b**) γ = −0.5, c = −0.2; (**c**) γ = −0.5, c = −0.5; (**d**) γ = −0.5, c = −0.8; (**e**) γ = −0.5, c = −1.2; (**f**) γ = −0.5, c = −1.8.

The stress concentration factor of FGM are given in Figure 7 when Young’s modulus varies in form of power-law $E\left(\phi \right)=E_{0}\left[1+\gamma {\left(\phi /a\right)}^{c}\right]$, where c<0. Figure 7a depicts the variation of Young’s modulus $E/E_{ref}$ with ϕ/a under different gradient control parameters where γ=−0.5,0,1.0 and c=1,2,3. When γ=0, it corresponds a homogeneous panel. When γ>0, with the increase of ϕ/a, $E/E_{ref}$ gradually decreases and finally tends to 1. When γ<0, with the increase of ϕ/a, $E/E_{ref}$ gradually increases and finally tends to 1. It can be seen that when ϕ/a is large enough, the γ and c have little influence on $E/E_{ref}$. Figure 7b shows the curve of stress concentration factor K for the r-FGM. As shown in Figure 7b, when the value of γ is positive, K increases significantly with the increase of the absolute value of *c*. When the value of γ is negative, K first decreases and then increases with the increase of the absolute value of *c*. The analysis shows that when γ<0, the stress concentration factor can be reduced compared with that of homogeneous materials. Figure 8 shows the dimensionless Von Mises stress distribution when γ=−0.5 which corresponds to the smallest optimal value of the stress concentration factor. It can be seen that the maximum value of the dimensionless Von Mises stress shows a significant decrease with the increase of *c*.

### 3.3. Failure Index and Damage Factor

#### 3.3.1. The Power-Law Inhomogeneous Variation When c > 0

**Figure 9:**
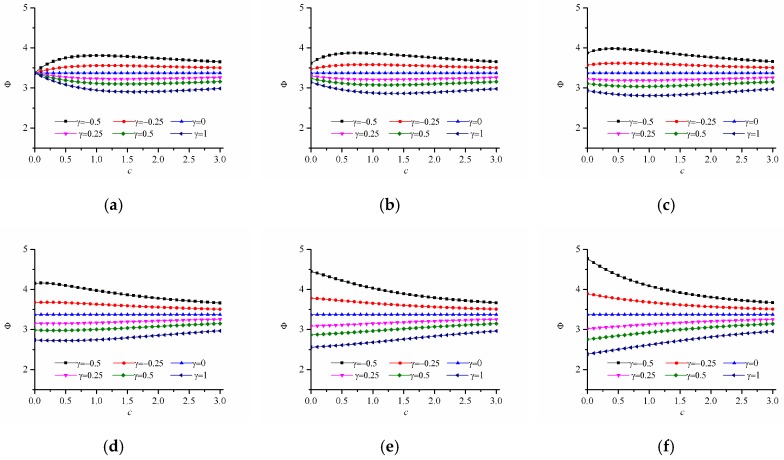
Variation of the failure index with different strength-modulus exponent ratio for the power-law inhomogeneous variation when c > 0. (**a**) δ=0; (**b**) δ=0.1; (**c**) δ=0.2; (**d**) δ=0.3; (**e**) δ=0.4; (**f**) δ=0.5.

**Figure 10:**
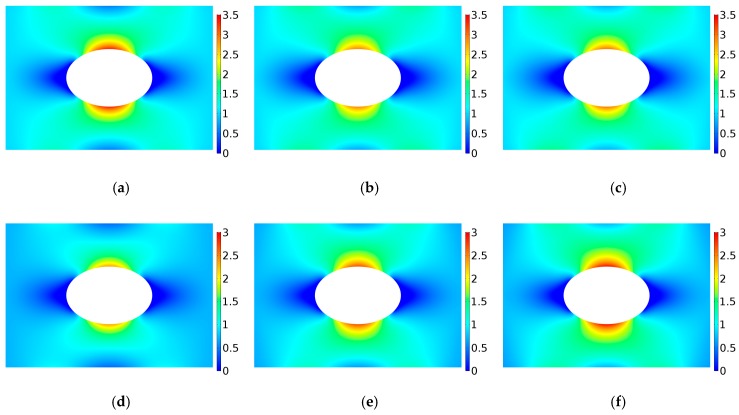
Variation of the damage factor ψ with power-law index under optimal condition of γ=1. (**a**) δ = 0.0, γ = 1, c = 0.0; (**b**) δ = 0.0, γ = 1, c = 1.5; (**c**) δ = 0.0, γ = 1, c = 3.0; (**d**) δ = 0.5, γ = 1, c = 0.0; (**e**) δ = 0.5, γ = 1, c = 1.5; (**f**) δ = 0.5, γ = 1, c = 3.0.

Figure 9 shows the calculation results of the failure index of r-FGM with hole under uniaxial tension with different strength-modulus exponent ratio when Young’s modulus varies in the power-law form as $E\left(\phi \right)=E_{0}\left[1+\gamma {\left(\phi /Lg\right)}^{c}\right]$, where c>0, and γ=−0.5,−0.3,−0.1,0.2,0.5,1.0. When this parameters are selected, the modulus of each point of the FGM satisfies $E\left(\phi \right)\in \left[E_{0}\left(1+\gamma \right),E_{0}\right]$ when γ < 0, and $E\left(\phi \right)\in \left[E_{0},E_{0}\left(1+\gamma \right)\right]$ when γ > 0. As can be seen from Figure 9, the failure index Φ, which is the maximum value of the damage factor, always decreases with the increasing modulus ratio γ. In addition, when δ<0.2, for the case of γ=1, the failure index Φ decreases rapidly and then tends to stabilize as the index of power-law increases. However, when δ>0.3, for the situation of γ=1, the failure index Φ shows a monotonously increasing trend as the index of power-law increases. Figure 10 shows the trend of the dimensional damage factor ψ with the index of power-law under optimal condition of γ=1. It can be clearly seen that when δ=0, the area of the FGM panel susceptible to damage (corresponding to the area shown by red in Figure 10a increases first and then decreases as the index of power-law increases, which is consistent with the result of γ=1 in Figure 9a. And the value of the dimensional damage factor is minimized when c = 1.5, which means that the optimal anti-failure performance of FGM is achieved. When δ=0.5, the damage factor increases slightly with the increasing power-law index, which is consistent with the result of γ=1 in Figure 9f.

**Figure 11:**
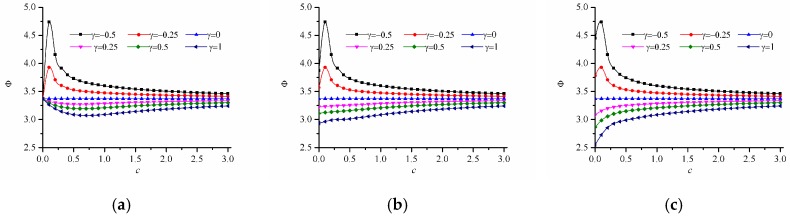

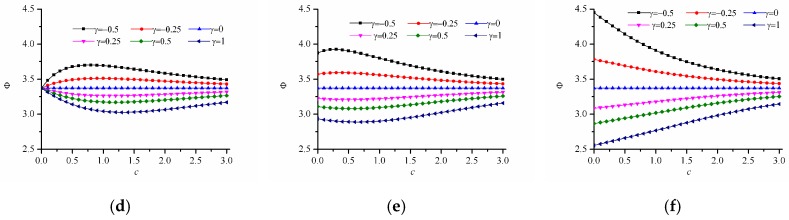
Variation of the failure index with different strength-modulus exponent ratio for the power-law inhomogeneous variation of x-FGM and y-FGM when c > 0. x-FGM: (**a**) δ=0.0; (**b**) δ=0.2; (**c**) δ=0.4; y-FGM: (**d**) δ=0.0; (**e**) δ=0.2; (**f**) δ=0.4.

Similarly, the variations of the failure index with different strength-modulus exponent ratio for the x-FGM and y-FGM are given in Figure 11. Here we still choose γ=−0.5,−0.3,−0.1,0.2,0.5,1.0, which makes the material modulus at each point satisfy max{E(ϕ)}/min{E(ϕ)}<2. From Figure 11, it can be seen that, for the elliptical hole, the trend curves of r-FGM and y-FGM are basically the same. In addition, it can be seen that when δ=0, the failure index changes with the index of power-law, there is an optimal functional gradient design function. When δ>0.2, the failure index increases with the index of power-law, which means that the optimal design modulus design is to select a uniform material that maximizes the modulus at each point. This phenomenon is because the maximum allowable stress of the material is a function of strength-modulus exponent ratio and modulus. When the strength-modulus exponent ratio is small, the change of the material modulus has little effect on the limit strength. The optimal design is to reduce the absolute stress at each point by adjusting the material modulus distribution. When the strength-modulus exponent ratio is large, increasing the material modulus causes the corresponding limit strength to increase rapidly, and then the damage factor at each point can be rapidly reduced. So, when δ>0.2 the solution is to select a uniform material that maximizes the modulus at each point.

#### 3.3.2. The Power-Law Inhomogeneous Variation When c < 0

**Figure 12:**
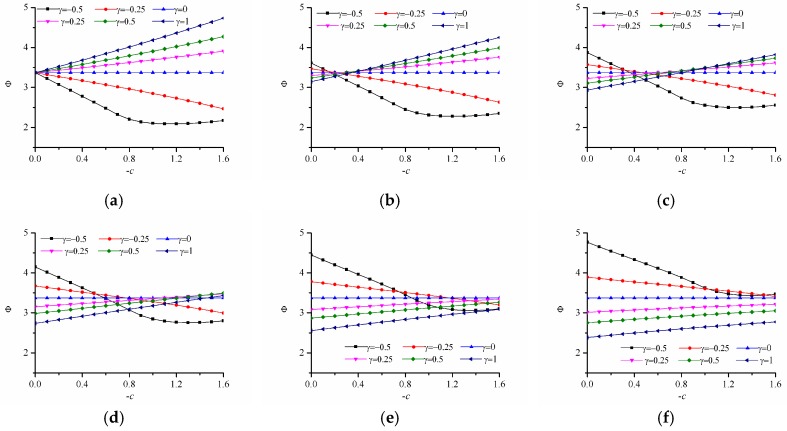
Variation of the failure index with different strength-modulus exponent ratio for the power-law inhomogeneous variation of r-FGM when c < 0. (**a**) δ=0; (**b**) δ=0.1; (**c**) δ=0.2; (**d**) δ=0.3; (**e**) δ=0.4; (**f**) δ=0.5.

Here, the failure index of FGM is calculated when Young’s modulus varies in form of power-law as $E\left(\phi \right)=E_{0}\left[1+\gamma {\left(\phi /a\right)}^{c}\right]$, where c<0. Here we still choose the parameter γ=−0.5,−0.3,−0.1,0.2,0.5,1.0, because this makes the material modulus at each point satisfy max{E(ϕ)}/min{E(ϕ)}<2. From Figure 12a–d, when δ<0.3 the failure index reaches a minimum value when γ=−0.5, and the material damage resistance is maximized. However, from Figure 12e,f, when δ>0.3, the failure index reaches a minimum at γ=1, where the material damage resistance is maximized. This optimization result can still be interpreted as the result of competition between the reduced stress value and the increase of the limit strength value. As shown in Figure 7a, this optimization result still shows that when δ<0.3, the optimal design is the modulus increasing with distance from the hole, and when δ>0.3, the optimal design is to maximize the modulus at each point.

## 4. Conclusions

In this paper, the effect of the inhomogeneous variation and gradient directions on stress concentration caused by a central elliptical hole in FGM panel under uniaxial tension load is analyzed. The effects of inhomogeneous characteristic control parameters, such as modulus ratio, the index of power-law variation is considered. The conclusions can be given as follows: (1) When the index of power-law variation is positive, the stress concentration factor of FGM can be reduced compared with that of homogeneous materials. (2) When the tensile load is along the x axis, the optimal designs of r-FGM significantly better than that of x-FGM and y-FGM. (3) When the associated variation of ultimate stress is considered, the choice of scheme to reduce the failure index is related to the strength-modulus exponent ratio. When the strength-modulus exponent ratio is small, the failure index changes with the index of power-law, which means there is an optimal FGM design. But when the strength-modulus exponent ratio is large, the optimal design modulus design is to select a uniform material that maximizes the modulus at each point. These research results have a certain reference value for further in-depth understanding of the inhomogeneous design for FGM.

## Figures and Tables

**Table 1:** The values of simulation parameters.

Values				
Simulations Parameters	Width of Rectangle Panel *W*	Length of Rectangle Panel *L*	Semi-Major Axis of Elliptical Hole *a*	Semi-Minor Axis of Elliptical Hole *b*
Figure 2	200 mm	*L*/*W* changes from 1 to 5	*a*/*W* changes from 0.05 to 0.4	*b* = *a*
Figure 3	200 mm	200 mm	Case for no hole, *b* = *a* = 0 mm	
Figure 4	200 mm	200 mm	10 mm	*b* = *a*
Figure 5, Figure 6, Figure 7, Figure 8, Figure 9, Figure 10, Figure 11 and Figure 12	200 mm	300 mm	60 mm	40 mm
